# Multi-center experience with outpatient total hip arthroplasty via a standard posterolateral approach

**DOI:** 10.1371/journal.pone.0292003

**Published:** 2024-03-14

**Authors:** Thomas D. Smith, Ian R. Wilson, Colin Burnell, James Vernon, David R. Hedden, Thomas R. Turgeon

**Affiliations:** 1 Faculty of Medicine, Dalhousie University, Halifax, Nova Scotia, Canada; 2 Department of Orthopaedics, University of British Columbia, Vancouver, British Columbia, Canada; 3 Department of Surgery, Rady Faculty of Medicine, University of Manitoba, Winnipeg, Manitoba, Canada; 4 Fort Whyte Orthopedics, Winnipeg, Manitoba, Canada; Assiut University Faculty of Medicine, EGYPT

## Abstract

**Background:**

This study sought to evaluate the safety, efficacy, and resource utilization of a pilot outpatient surgery program for total hip arthroplasty compared to traditional inpatient total hip arthroplasty performed via the posterolateral approach.

**Methods:**

A cohort of 68 patients from two sites were enrolled in a regional pilot project for outpatient total hip arthroplasty (THA) and matched 1:1 against a cohort of patients undergoing routine inpatient THA. Data was extracted retrospectively from patient and hospital charts including adverse events (AE), readmission within 90 days, emergency room (ER) visits, patient calls, patient-reported outcome measures, length of stay, and multiple surgical variables.

**Results:**

The outpatient group had a mean hospital stay of 13 hours, whereas the inpatient group had a mean of 58 hours (p<0.001). Three outpatients and four inpatients experienced post-op complications. Three inpatients and one outpatient visited the ER within 8 weeks of surgery. No difference in pre-operative hemoglobin (p = 0.210), or surgical blood loss (p = 0.550) was found between study groups. There was no difference found between groups regarding Oxford-12 Hip Score improvement, nor satisfaction at six months, one and two years (p>0.125).

**Conclusion:**

This study demonstrates that outpatient THA using the posterolateral approach is as safe and effective as inpatient THA for overall healthy and carefully screened patients, based on the low rate of AEs observed and similar patient outcomes reported. Significantly reduced time in hospital demonstrates the reduced healthcare resources associated with outpatient THA.

## Introduction

Total hip arthroplasty (THA) is a commonly performed procedure with a high rate of success and patient satisfaction, favourable long-term implant survivorship, demonstrated cost-effectiveness, and an established safety profile [1–3].

The length of stay (LOS) after THA has steadily declined over time, from weeks down to hours after surgery [4–9]. THA’s evolution to an outpatient procedure stems from multiple factors including, improved surgical techniques with decreased invasiveness, pain control optimization, comprehensive patient education and expectation management, advances in blood conservation strategy, as well as the development of protocols to mitigate risk, emphasize safety, and select appropriate surgical candidates [4–10].

Financial considerations have also driven the transition of THA to outpatient surgery. Whether in an overburdened public health system with budgetary constraints or a private third-party payer model with bundled payments, policy-makers and stakeholders are continually looking to maximize efficiency and reduce costs, where possible, while minimizing the effects on safety, readmissions, quality of care, and the overall patient experience [11–15].

Minimally invasive and anatomy sparing surgical techniques are often cited as primary reasons for the increasing prevalence of outpatient THA [10, 16]. The anterior approach [10, 11, 17–20], minimally invasive posterior approach [12, 21–23], or two-incision approach [24, 25] are commonly utilized in outpatient THA studies, with the standard posterolateral approach used less frequently [26–29]. This appears to be the case despite no clear advantage in outcomes of the anterior approach compared to the posterolateral approach [30, 31].

The primary purpose of this retrospective study was to evaluate the safety, efficacy, resource utilization and outcomes of a pilot program for outpatient THA compared to a matched cohort of traditional inpatient THA performed utilizing the standard posterolateral approach. To our knowledge, this study is the first of its kind in Canada that utilizes the posterolateral approach and is amongst a small cohort of studies from the United States, Europe, and worldwide that use this surgical approach in the context of outpatient hip arthroplasty [26, 27, 29].

## Methods

Between September 2017 and December 2018, 68 consecutive primary cementless THAs were performed via a standard posterolateral approach as part of a local outpatient THA pilot program. All patients enrolled provided written consent to have their data used for research purposes. We obtained ethics approval from the University of Manitoba Bannatyne Campus Research Ethics Board (H2018:141) and the Winnipeg Regional Health Authority before data collection and accessed the data September-November, 2021.

We defined outpatient surgery as a procedure followed by discharge from hospital without a planned overnight stay. Data collected included patient demographics such as age, gender, body mass index (BMI), preoperative hemoglobin, American Society of Anaesthesiologists (ASA) score, and patient comorbidities, as well as surgical factors such as surgical duration and estimated blood loss (EBL). Adverse events such as failure to discharge, 90-day readmission, ER visits, and medical complications outside the expected post-surgical course were also recorded from the patient hospital and office charts. We recorded Oxford Hip Score pre-operatively and at 6 months and 1 year post-operatively at one of our two participating institutions as well as a 5-point Likert satisfaction score (very satisfied, satisfied, neutral, unsatisfied, very unsatisfied) at six months and one year postoperatively. Outpatients were matched by sex, age, and BMI to a cohort of patients undergoing routine inpatient THA.

### Patient selection

Patients from the local outpatient pilot project were used for this study and matched to a cohort of patients from an existing database that had undergone a routine hospital stay after THA between January 2016 and March 2020. Matching criteria were exact gender, age +/- 4 years, BMI +/- 10, and number of comorbidities +/- 2. Inclusion criteria for the outpatient group comprised radiographic findings of osteoarthritis, a BMI less than 40, ASA score less than 2, an absence of cardiopulmonary disease that would require inpatient monitoring after surgery, independent ambulation without a mobility aid, a preoperative hemoglobin greater than 120 g/L for women and greater than 130 g/L for men, and a willingness to participate in the outpatient protocol including attendance of educational classes as described below. Patients were required to live within 60 minutes of the operative hospital and 30 minutes of an emergency department. Patients also required assistance at home–either by family or friends–for a minimum of 2 days postoperatively. Ultimately, surgical and anesthetic consultants agreed upon a specific surgical candidates’ participation in the program.

Patients were excluded from the outpatient group if they had a history of chronic opioid or benzodiazepine use. Patients taking anticoagulants other than acetylsalicylic acid or excessive alcohol use were also excluded.

All selected outpatients participated in a preoperative education class and were taught the standard post-operative exercise program prior to admission. Patients were required to attend all follow up appointments, obtain recovery equipment such as a cane, and walker, as well as discharge medication in advance of surgery. Discharge medication included prophylactic antibiotics, pain medication, antiemetics, and deep venous thrombosis prophylaxis with low dose ASA. Inpatients were offered non-mandatory and less-extensive preoperative education classes and were sent home with the standard post-operative exercise program prior to discharge. Discharge medication included pain medication and deep venous thrombosis prophylaxis scaled to the patient’s medical risk. Routine outpatient physiotherapy was not utilized.

### Outpatient surgical protocol

Patients included for the pilot project were scheduled as the first case of the day. Five surgeons performed procedures at two separate facilities. Standard preoperative medications for both groups included a long-acting hydromorphone, gabapentin, celecoxib, acetaminophen and ondansetron per the discretion of the anesthesiologist. All patients were administered intravenous prophylactic antibiotics and tranexamic acid. Patients received spinal anesthesia with 0.5–0.75% bupivacaine dosed based on expected surgical time and patient mass with sedation at the discretion of the anesthesiologist. A cementless THA was performed through a standard posterolateral approach in all patients. Standard closure, periarticular injection and dressings were applied.

Postoperatively, standard patient vitals were monitored, and diet was progressed. Oral medications were administered for pain and nausea as needed. Mobilisation including transfers, walking and stairs were performed under the supervision of nursing and physiotherapy. A second oral dose of tranexamic acid was administered prior to discharge for outpatients. A portable post-operative anteroposterior pelvic radiograph was evaluated by the attending surgeon prior to discharge.

Discharge criteria were met when patients could mobilize and perform activities of daily living such as dressing, eating, and toileting independently or with the aid of support via a walker or crutches as determined by therapists and nursing based on regional protocols. As this was a pilot project, an inpatient bed was reserved overnight in case of admission.

### Follow up

All patients in the outpatient surgery arm were contacted by a team member post operative day one to ensure patient stability. Subsequently, all patients had follow-up at two and six weeks, six months, one year and two years for clinical assessment. Patient reported outcome measures (PROMs) in the form of a feedback questionnaire including a 5-point Likert satisfaction score and Oxford Hip Scores (OHS) were administered at six months, one year and two years post-operatively.

### Statistical analysis

Statistical Analysis Software (v9.4, Cary, North Carolina) was used to compare groups using *t*-test, Wilcoxon rank sums, and Fischer’s exact test. Satisfaction was coded as either satisfied (very satisfied or satisfied) or dissatisfied (neutral, unsatisfied, very unsatisfied). A p-value < 0.05 indicated statistical significance.

## Results

### Preoperative

The medical records of 68 patients who underwent outpatient THA were reviewed and data was extracted and compared to a 1:1 matched cohort whom underwent inpatient THA. As expected, there were no statistical differences in the matching variables of age, BMI, number of comorbidities, or gender between groups (Table 1). There was a small, but statistically significant difference in ASA scores between outpatients and inpatients (ASA 1.6 vs 2.0, p<0.001). Pre-operative Oxford Hip Scores did not differ between outpatients and inpatients (21.5 vs 19.3, p = 0.13,) nor did pre-operative hemoglobin (145 vs 142g/L, p = 0.27).

**Table 1:** Preoperative statistics.

	Outpatient		Inpatient		
	N	Mean (SD)	N	Mean (SD)	P-value (test)
Age	68	54.8 (7.2)	68	54.9 (7.0)	0.96 (t-test)
Gender	28F 40M		28F 40M		N/A
BMI	42	28.5 kg/m^2^ (4.2)	58	28.9 kg/m^2^ (4.9)	0.68 (t-test)
Comorbidities, number	68	2.7 (1.6)	22	2.5 (1.2)	0.059 (t-test)
ASA	64	1.6 (0.5)	62	2.0 (0.5)	<0.001 (Wilcoxon)
Oxford Hip Score, Pre-op	42	21.5 (8.1)	65	19.3 (6.8)	0.13 (t-test)
Pre-op Hemoglobin	68	145.1 (12.5)	55	142.5 (13.2)	0.27 (t-test)

### Perioperative

Surgical time was significantly shorter for outpatients compared to inpatients (45.5 vs 78.5 minutes, p<0.001; Table 2). Blood loss did not differ between outpatient and inpatient groups (314 vs 328 mL, p = 0.55) and no inpatients, nor outpatients received a blood transfusion. Mean total hospital length of stay was 13 hours for the outpatient group and 58 hours for the inpatient group. Among the outpatient group, seven failed discharge (10%): two due to hypotension limiting mobility, one due to pain control, one due to significant wound drainage, one due to urinary retention and two due to undiagnosed obstructive sleep apnea (OSA).

**Table 2:** Perioperative patient metrics.

	Outpatient		Inpatient		
	N	Mean (SD)	N	Mean (SD)	P value (test)
Total hospital length of stay (hours)	67	13.0 (7.6)	68	58.3 (30.5)	<0.001 (t-test)
Surgery Time (mins)	68	45.5 (9.8)	68	78.3 (19.0)	<0.001 (t-test)
Blood Loss (mL)	66	314 (141)	67	328 (140)	0.55 (t-test)

### Postoperative

#### Patient reported outcomes

At six months, one year, and two years postoperatively, there were no differences in Oxford-12 Hip Scores or change from pre-operative scores between the two groups (Table 3, Postoperative Statistics). Satisfaction did not differ between groups at six months, one year, or two years.

**Table 3:** Patient complications, Oxford Hip Scores and satisfaction post-surgery.

	Outpatient		Inpatient		
	N	Mean (SD)	N	Mean (SD)	P-value (test)
Complications/ER Visits post-discharge	68	3	68	4	1 (Fisher’s Exact)
Mortality (30 day)	68	0	68	0	N/A
Oxford Hip, 6 month	40	44.0 (4.3)	56	43.4 (5.5)	0.53 (t-test)
Oxford Hip, Improvement 6M	39	22.8 (9.0)	56	24.3 (7.1)	0.38 (t-test)
Oxford Hip, 12 month	41	45.3 (4.6)	55	43.6 (6.2)	0.13 (t-test)
Oxford Hip, Improvement pre-op to 12M	41	24.2 (8.4)	55	24.6 (7.4)	0.79 (t-test)
Oxford Hip, 24 month	30	44.1 (7.4)	35	45.5 (3.8)	0.32 (t-test)
Oxford Hip Improvement pre-op to 24M	30	23.1 (9.2)	35	26.2 (6.5)	0.12 (t-test)
Satisfaction 6 month	40	38 Satisfied 2 Dissatisfied	56	52 Satisfied 4 Dissatisfied	0.31 (Fisher’s Exact)
Satisfaction 12 month	41	40 Satisfied 1 Dissatisfied	55	55 Satisfied	0.43 (Fisher’s Exact)
Satisfaction 24 month	29	29 Satisfied	33	32 Satisfied 1 Dissatisfied	0.53 (Fisher’s Exact)

#### Complications

Amongst the outpatient group, seven subjects were unable to discharge home on the same day requiring at least one overnight stay. Two patients had symptomatic hypotension, two had undiagnosed OSA and one had poor pain control. All of these events were considered as expected sequelae of surgery and were not deemed to be complications from treatment. Two additional patients required at least one overnight stay due to urinary retention and wound drainage, that was ultimately treated with oral antibiotics, respectively. An additional outpatient sought treatment for urinary retention in the emergency room following discharge, but was not readmitted. Despite careful chart review for all patients, no complications were documented during the initial hospital stay for any of the inpatient controls. Four inpatients did return to the emergency room within 90 days. One patient was readmitted for pain control within three weeks of surgery. One was admitted and underwent revision surgery for infection within five weeks. One patient presented to the ER with dizziness and blood pressure control concerns. A fourth patient attended the ER within two days of discharge, but the reason for the visit is unclear from the chart documentation. No statistical difference was seen in the combined in-hospital complications and ER visits between groups (p = 1).

## Discussion

This study demonstrates that outpatient THA is achievable via the standard posterolateral approach in appropriately selected candidates without compromising patient safety. Of the 68 participants in the outpatient group, all but seven were able to be discharged within hours after surgery with none requiring readmission to hospital. There were no differences found in complication incidence between groups. Further, PROMs such as satisfaction and OHS with the outpatient program improved significantly and were not inferior to the standard group of inpatients.

Outpatient arthroplasty is not a new concept with literature well established in both hip and knee arthroplasty [14]. Much of the literature on outpatient THA includes use of the anterior approach and generally have shown safety and equivalent PROMs to traditional inpatient procedures [10, 18]. In contrast, studies that utilize the minimally invasive or standard posterolateral approach are few in number, have a short length of follow-up, and rarely incorporate PROMs [12, 22, 23, 26–29].

In our study, 61 of 68 patients (90%) were discharged safely and successfully on the same day as their surgery. This discharge rate is consistent with other studies of outpatient THA regardless of approach [10, 12, 14, 17, 18, 21–29, 32].

Further, reasons for discharge failure in our study group, other than obstructive sleep apnea, are also common among other outpatient THA studies, regardless of surgical approach [14, 32, 33]. The two patients in our study with undisclosed or suspected obstructive sleep apnea were admitted out of a concern for safety and perhaps highlights the need for a proactive approach to patient selection.

There were no readmissions to hospital in our study cohort. Keeping the readmission rate low is important to the viability of outpatient THA protocols as the cost savings on behalf of both the patient and hospital diminish rapidly if there is a readmission [5, 17, 29, 32, 33]. A review by Chambers *et al*. identified strategies to reduce readmissions including an emphasis on appropriate patient selection and presurgical optimization, efficient care protocols, appropriate and timely management of complications. [5]. Multiple review studies have shown common risk factors for failure of same-day discharge including age greater than 65, ASA score greater than 2, and comorbidities such as cardiovascular disease, renal disease, diabetes mellitus, malnutrition, or active smoking [14, 32, 33]. Our protocols and selection criteria factored in many of these strategies and risk factors and may explain our high percentage of successful discharges and lack of readmissions.

The reported incidence of complications after outpatient THA (n = 3) was low in our patient group and there was no difference seen between the two cohorts. Multiple studies have demonstrated similar complication rates between outpatient and inpatient THA [10, 14, 32]. Our results suggest that safety is independent of surgical approach if effective strategies and protocols are implemented and known risk factors for complication are considered in patient selection.

In addition to demonstrating data on safety of outpatient THA, we showed that there was no difference between the outpatient and inpatient THA cohorts regarding the measured PROMs including Oxford-12 Hip Score at all time points out to two years. Mean OHS improvement was similar between study groups at over 20 points. Satisfaction between groups did not differ significantly between groups at any point. The use of PROMs to date in the outpatient THA literature has been sparse, with only a few studies incorporating them into their results [18, 19, 22, 28]. Our study adds to the emerging literature of PROMs in the outpatient literature and further demonstrates outcomes similar to traditional inpatient procedures.

A significant difference was found between the outpatient and inpatient durations of surgery with inpatient procedures taking a mean of 33min longer than the outpatients. All outpatients were the first case of the day and met a number of criteria beyond those used to match as not all were documented in the inpatient control group. This may have led to higher complexity of the cases which could translate into higher complication rates in the inpatient group. Despite this, the complication rate in the inpatient group was quite low suggesting little evidence of this concern. Additionally, outpatient cases were performed consistently by attending surgeons while an unknown number of the control group cases would have been performed with a learner performing some or all of the case with the attending present. This too would tend to lead to longer OR times in the control group without necessarily affecting the complication rate.

The main limitation of this study is the relatively small outpatient cohort. While comparable to several studies of a similar nature, selection of appropriate patients is integral to the success of an outpatient arthroplasty program and strict adherence to appropriate inclusion criteria is valued over inflating participant numbers and potentially compromising patient safety. As various facets of the protocol continue to be refined, the number of eligible candidates may increase and is a possibility for future study. We were unable to perform an *a priori* power analysis as there was insufficient data in the literature to perform one when we initiated this study. The process of patient enrollment also highlights the selection bias that is inherent in outpatient arthroplasty, yet this is a necessary limitation. In general, younger and healthier patients with fewer comorbidities and a lower ASA score are considered acceptable candidates. An additional limitation is a lack of costing and financial analysis. While cost savings are implied due to the nature of outpatient arthroplasty and reduced length of hospital stay, and this fact has been confirmed via other studies to date, we did not set out to determine this specifically with the present study. Though this would be ideal to include in future research, costing is often difficult to achieve and, therefore, demonstrating safety of surgery performed using limited resources such as inpatient hospital infrastructure is meaningful.

While we did include a cohort group propensity matched via multiple factors, there was a significant difference in ASA score between the outpatient and inpatient groups and is likely a result of our non-randomized design. Despite the statistical significance of this, it remains unclear whether this constitutes a relevant clinical difference as the number of comorbidities was similar between groups. Finally, further limitations include a lack of randomization, and a lack of generic PROMs being utilized at all participating institutions. Due to the retrospective nature of this study, it was not possible to fill in gaps in missing data at the time of data analysis, which limits the statistical power of our analysis and may have led to bias in our results. It is possible that these factors combined led to a residual bias towards worse outcomes for inpatients in this study.

## Conclusion

To our knowledge, this is the first Canadian study that demonstrates safety and efficacy of an outpatient THA program using the standard posterolateral approach. We demonstrated acceptably low complications with no readmissions and improvements in PROMs and a high level of patient satisfaction for overall healthy and carefully screened patients in keeping with other studies. This adds to the mounting evidence of safety in outpatient total joint arthroplasty. Studies including randomized control trials are needed to refine appropriate selection criteria and protocols. We believe outpatient total hip arthroplasty using the standard posterolateral approach is a safe and effective strategy to provide care.

## Supporting information

S1 Checklist *PLOS ONE* clinical studies checklist. (DOCX)

S2 Checklist STROBE statement—checklist of items that should be included in reports of observational studies. (DOCX)

## References

[1] Garellick G, Malchau H, Herberts P. Survival of hip replacements. A comparison of a randomized trial and a registry. Clin Orthop Relat Res. 2000(375):157–67. 10853165

[2] Jenkins PJ, Clement ND, Hamilton DF, Gaston P, Patton JT, Howie CR. Predicting the cost-effectiveness of total hip and knee replacement: a health economic analysis. Bone Joint J. 2013;95-B(1):115–21. doi: 10.1302/0301-620X.95B1.29835 23307684

[3] Kärrholm JL H; Malchau H; Mohaddes M; Nemes S; Rogmark C; Rolfson O. The Swedish Hip Arthroplasty Register Annual Report 2016. The Swedish Hip Arthroplasty Register. 2016.

[4] Aggarwal V, Thakkar S, Collins K, Vigdorchik J. Same Day Discharge After Total Joint Arthroplasty The Future May Be Now. Bull Hosp Jt Dis (2013). 2017;75(4):252–6. 29151010

[5] Chambers MC, El-Othmani MM, Anoushiravani AA, Sayeed Z, Saleh KJ. Reducing 30-day Readmission After Joint Replacement. Orthop Clin North Am. 2016;47(4):673–80. doi: 10.1016/j.ocl.2016.05.014 27637653

[6] Foote J, Panchoo K, Blair P, Bannister G. Length of stay following primary total hip replacement. Ann R Coll Surg Engl. 2009;91(6):500–4. doi: 10.1308/003588409X432356 19558767 PMC2966203

[7] Husted H, Hansen HC, Holm G, Bach-Dal C, Rud K, Andersen KL, et al. What determines length of stay after total hip and knee arthroplasty? A nationwide study in Denmark. Arch Orthop Trauma Surg. 2010;130(2):263–8. doi: 10.1007/s00402-009-0940-7 19633865

[8] Ramkumar PN, Chu CT, Harris JD, Athiviraham A, Harrington MA, White DL, et al. Causes and Rates of Unplanned Readmissions After Elective Primary Total Joint Arthroplasty: A Systematic Review and Meta-Analysis. Am J Orthop (Belle Mead NJ). 2015;44(9):397–405. 26372748

[9] Walters M, Chambers MC, Sayeed Z, Anoushiravani AA, El-Othmani MM, Saleh KJ. Reducing Length of Stay in Total Joint Arthroplasty Care. Orthop Clin North Am. 2016;47(4):653–60. doi: 10.1016/j.ocl.2016.05.006 27637651

[10] Richards M, Alyousif H, Kim JK, Poitras S, Penning J, Beaule PE. An Evaluation of the Safety and Effectiveness of Total Hip Arthroplasty as an Outpatient Procedure: A Matched-Cohort Analysis. J Arthroplasty. 2018;33(10):3206–10. doi: 10.1016/j.arth.2018.05.036 29914820

[11] Aynardi M, Post Z, Ong A, Orozco F, Sukin DC. Outpatient surgery as a means of cost reduction in total hip arthroplasty: a case-control study. HSS J. 2014;10(3):252–5. doi: 10.1007/s11420-014-9401-0 25264442 PMC4171452

[12] Bertin KC. Minimally invasive outpatient total hip arthroplasty: a financial analysis. Clin Orthop Relat Res. 2005(435):154–63. doi: 10.1097/01.blo.0000157173.22995.cf 15930933

[13] Huang A, Ryu JJ, Dervin G. Cost savings of outpatient versus standard inpatient total knee arthroplasty. Can J Surg. 2017;60(1):57–62. doi: 10.1503/cjs.002516 28234591 PMC5373744

[14] Pollock M, Somerville L, Firth A, Lanting B. Outpatient Total Hip Arthroplasty, Total Knee Arthroplasty, and Unicompartmental Knee Arthroplasty: A Systematic Review of the Literature. JBJS Rev. 2016;4(12). doi: 10.2106/JBJS.RVW.16.00002 28060788

[15] Meding JBB M. E. Outpatient total joint arthroplasty: Is the paradigm changing? Seminars in Arthroplasty. 2015;26:210–2.

[16] Bodrogi A, Dervin GF, Beaule PE. Management of patients undergoing same-day discharge primary total hip and knee arthroplasty. CMAJ. 2020;192(2):E34–E9. doi: 10.1503/cmaj.190182 31932338 PMC6957327

[17] Fraser JF, Danoff JR, Manrique J, Reynolds MJ, Hozack WJ. Identifying Reasons for Failed Same-Day Discharge Following Primary Total Hip Arthroplasty. J Arthroplasty. 2018;33(12):3624–8. doi: 10.1016/j.arth.2018.08.003 30172415

[18] Goyal N, Chen AF, Padgett SE, Tan TL, Kheir MM, Hopper RH Jr., et al. Otto Aufranc Award: A Multicenter, Randomized Study of Outpatient versus Inpatient Total Hip Arthroplasty. Clin Orthop Relat Res. 2017;475(2):364–72. doi: 10.1007/s11999-016-4915-z 27287858 PMC5213925

[19] Hartog YM, Mathijssen NM, Vehmeijer SB. Total hip arthroplasty in an outpatient setting in 27 selected patients. Acta Orthop. 2015;86(6):667–70. doi: 10.3109/17453674.2015.1066211 26139431 PMC4750764

[20] Van Den Eeden YN, De Turck BJ, Van Den Eeden FM. 24 hours stay after hip replacement. Acta Orthop. 2017;88(1):24–8. 27658640 10.1080/17453674.2016.1236229 PMC5251260

[21] Dawson-Bowling SJ, Jha S, Chettiar KK, East DJ, Gould GC, Apthorp HD. A multidisciplinary enhanced recovery programme allows discharge within two days of total hip replacement; three- to five-year results of 100 patients. Hip Int. 2014;24(2):167–74. doi: 10.5301/hipint.5000100 24500823

[22] Dorr LD, Thomas DJ, Zhu J, Dastane M, Chao L, Long WT. Outpatient total hip arthroplasty. J Arthroplasty. 2010;25(4):501–6. doi: 10.1016/j.arth.2009.06.005 19640672

[23] Klein GR, Posner JM, Levine HB, Hartzband MA. Same Day Total Hip Arthroplasty Performed at an Ambulatory Surgical Center: 90-Day Complication Rate on 549 Patients. J Arthroplasty. 2017;32(4):1103–6. doi: 10.1016/j.arth.2016.10.013 27890310

[24] Berger RA, Kusuma SK, Sanders SA, Thill ES, Sporer SM. The feasibility and perioperative complications of outpatient knee arthroplasty. Clin Orthop Relat Res. 2009;467(6):1443–9. doi: 10.1007/s11999-009-0736-7 19238499 PMC2674174

[25] Berger RA. Total hip arthroplasty using the minimally invasive two-incision approach. Clin Orthop Relat Res. 2003(417):232–41. doi: 10.1097/01.blo.0000096828.67494.95 14646722

[26] Gromov K, Jorgensen CC, Petersen PB, Kjaersgaard-Andersen P, Revald P, Troelsen A, et al. Complications and readmissions following outpatient total hip and knee arthroplasty: a prospective 2-center study with matched controls. Acta Orthop. 2019;90(3):281–5. doi: 10.1080/17453674.2019.1577049 30739559 PMC6534233

[27] Larsen JR, Skovgaard B, Pryno T, Bendikas L, Mikkelsen LR, Laursen M, et al. Feasibility of day-case total hip arthroplasty: a single-centre observational study. Hip Int. 2017;27(1):60–5. doi: 10.5301/hipint.5000421 27791240

[28] Madsen MN, Kirkegaard ML, Laursen M, Larsen JR, Pedersen MF, Skovgaard B, et al. Low complication rate after same-day total hip arthroplasty: a retrospective, single-center cohort study in 116 procedures. Acta Orthop. 2019;90(5):439–44. doi: 10.1080/17453674.2019.1637631 31274038 PMC6746288

[29] Springer BD, Odum SM, Vegari DN, Mokris JG, Beaver WB Jr. Impact of Inpatient Versus Outpatient Total Joint Arthroplasty on 30-Day Hospital Readmission Rates and Unplanned Episodes of Care. Orthop Clin North Am. 2017;48(1):15–23. doi: 10.1016/j.ocl.2016.08.002 27886679

[30] Graves SC, Dropkin BM, Keeney BJ, Lurie JD, Tomek IM. Does Surgical Approach Affect Patient-reported Function After Primary THA? Clin Orthop Relat Res. 2016;474(4):971–81. doi: 10.1007/s11999-015-4639-5 26620966 PMC4773324

[31] Higgins BT, Barlow DR, Heagerty NE, Lin TJ. Anterior vs. posterior approach for total hip arthroplasty, a systematic review and meta-analysis. J Arthroplasty. 2015;30(3):419–34. doi: 10.1016/j.arth.2014.10.020 25453632

[32] Basques BA, Tetreault MW, Della Valle CJ. Same-Day Discharge Compared with Inpatient Hospitalization Following Hip and Knee Arthroplasty. J Bone Joint Surg Am. 2017;99(23):1969–77. doi: 10.2106/JBJS.16.00739 29206786

[33] Courtney PM, Boniello AJ, Berger RA. Complications Following Outpatient Total Joint Arthroplasty: An Analysis of a National Database. J Arthroplasty. 2017;32(5):1426–30. doi: 10.1016/j.arth.2016.11.055 28034481

