# Supplementary material for: Multi-center experience with outpatient total hip arthroplasty via a standard posterolateral approach
Source: PLoS One. 2024 Mar 14;19(3):e0292003. doi: 10.1371/journal.pone.0292003 (PMC10939296; doi:10.1371/journal.pone.0292003)
Supplement: S2 Checklist — (DOCX) [file pone.0292003.s002.docx]

STROBE Statement—checklist of items that should be included in reports of observational studies

|  | Item No. | Recommendation | Page  No. | Relevant text from manuscript |
| --- | --- | --- | --- | --- |
| **Title and abstract** | 1 | (*a*) Indicate the study’s design with a commonly used term in the title or the abstract | 2 | “A cohort of” |
|  |  | (*b*) Provide in the abstract an informative and balanced summary of what was done and what was found | 2 | Full abstract |
| Introduction | | | |  |
| Background/rationale | 2 | Explain the scientific background and rationale for the investigation being reported | 3 | Full introduction |
| Objectives | 3 | State specific objectives, including any prespecified hypotheses | 3-4 | “The primary purpose of this retrospective study was to evaluate the safety, efficacy, resource utilization and outcomes of a pilot program for outpatient THA compared to a matched cohort of traditional inpatient THA performed utilizing the standard posterolateral approach.” |
| Methods | | | |  |
| Study design | 4 | Present key elements of study design early in the paper | 4-7 | Full methods |
| Setting | 5 | Describe the setting, locations, and relevant dates, including periods of recruitment, exposure, follow-up, and data collection | 4-7 | Full methods |
| Participants | 6 | (*a*) *Cohort study*—Give the eligibility criteria, and the sources and methods of selection of participants. Describe methods of follow-up  *Case-control study*—Give the eligibility criteria, and the sources and methods of case ascertainment and control selection. Give the rationale for the choice of cases and controls  *Cross-sectional study*—Give the eligibility criteria, and the sources and methods of selection of participants | 4-6 | “Patients from the local outpatient pilot project were used for this study and matched to a cohort of patients from an existing database that had undergone a routine hospital stay after THA between January 2016 and March 2020. ”  “All patients in the outpatient surgery arm were contacted by a team member post operative day one to ensure patient stability. Subsequently, all patients had follow-up at two and six weeks, six months, one year and two years for clinical assessment. Patient reported outcome measures (PROMs) in the form of a feedback questionnaire including a 5-point Likert satisfaction score and Oxford Hip Scores (OHS) were administered at six months, one year and two years post-operatively.” |
|  |  | (*b*) *Cohort study*—For matched studies, give matching criteria and number of exposed and unexposed  *Case-control study*—For matched studies, give matching criteria and the number of controls per case | 4-5 | “Matching criteria were exact gender, age +/- 4 years, BMI +/- 10, and number of comorbidities +/- 2.” |
| Variables | 7 | Clearly define all outcomes, exposures, predictors, potential confounders, and effect modifiers. Give diagnostic criteria, if applicable | 5-7 | Full methods |
| Data sources/ measurement | 8* | For each variable of interest, give sources of data and details of methods of assessment (measurement). Describe comparability of assessment methods if there is more than one group | *5-7* | *Full methods* |
| Bias | 9 | Describe any efforts to address potential sources of bias |  | See matching + active followup: “All patients in the outpatient surgery arm were contacted by a team member post operative day one to ensure patient stability. Subsequently, all patients had follow-up at two and six weeks, six months, one year and two years for clinical assessment” |
| Study size | 10 | Explain how the study size was arrived at | All patients, p. 4-5 | Patient selection piece |

Continued on next page

| Quantitative variables | 11 | Explain how quantitative variables were handled in the analyses. If applicable, describe which groupings were chosen and why | 4-7 | Full methods |
| --- | --- | --- | --- | --- |
| Statistical methods | 12 | (*a*) Describe all statistical methods, including those used to control for confounding | 7 | “Statistical Analysis Software (v9.4, Cary, North Carolina) was used to compare groups using t-test, Wilcoxon rank sums, and Fischer’s exact test. Satisfaction was coded as either satisfied (very satisfied or satisfied) or dissatisfied (neutral, unsatisfied, very unsatisfied). A p-value < 0.05 indicated statistical significance.” |
|  |  | (*b*) Describe any methods used to examine subgroups and interactions | N/A |  |
|  |  | (*c*) Explain how missing data were addressed | Excluded from analysis |  |
|  |  | (*d*) *Cohort study*—If applicable, explain how loss to follow-up was addressed  *Case-control study*—If applicable, explain how matching of cases and controls was addressed  *Cross-sectional study*—If applicable, describe analytical methods taking account of sampling strategy | Patients actively contacted |  |
|  |  | (*e*) Describe any sensitivity analyses | N/A |  |
| Results | | | | |
| Participants | 13* | (a) Report numbers of individuals at each stage of study—eg numbers potentially eligible, examined for eligibility, confirmed eligible, included in the study, completing follow-up, and analysed | 7 | “The medical records of 68 patients who underwent outpatient THA were reviewed and data was extracted and compared to a 1:1 matched cohort whom underwent inpatient THA. ” |
|  |  | (b) Give reasons for non-participation at each stage | N/A |  |
|  |  | (c) Consider use of a flow diagram | N/A |  |
| Descriptive data | 14* | (a) Give characteristics of study participants (eg demographic, clinical, social) and information on exposures and potential confounders | 7-8 | Full results section |
|  |  | (b) Indicate number of participants with missing data for each variable of interest | Tables | See tables |
|  |  | (c) *Cohort study*—Summarise follow-up time (eg, average and total amount) | N/A |  |
| Outcome data | 15* | *Cohort study*—Report numbers of outcome events or summary measures over time | *Tables* | *See tables* |
|  |  | *Case-control study—*Report numbers in each exposure category, or summary measures of exposure |  |  |
|  |  | *Cross-sectional study—*Report numbers of outcome events or summary measures |  |  |
| Main results | 16 | (*a*) Give unadjusted estimates and, if applicable, confounder-adjusted estimates and their precision (eg, 95% confidence interval). Make clear which confounders were adjusted for and why they were included | No regression |  |
|  |  | (*b*) Report category boundaries when continuous variables were categorized | No regression |  |
|  |  | (*c*) If relevant, consider translating estimates of relative risk into absolute risk for a meaningful time period | No regression |  |

Continued on next page

| Other analyses | 17 | Report other analyses done—eg analyses of subgroups and interactions, and sensitivity analyses | N/A |  |
| --- | --- | --- | --- | --- |
| Discussion | | | | |
| Key results | 18 | Summarise key results with reference to study objectives | 9-12 | Full discussion |
| Limitations | 19 | Discuss limitations of the study, taking into account sources of potential bias or imprecision. Discuss both direction and magnitude of any potential bias | 11 | “The main limitation of this study is the relatively small outpatient cohort. While comparable to several studies of a similar nature, selection of appropriate patients is integral to the success of an outpatient arthroplasty program and strict adherence to appropriate inclusion criteria is valued over inflating participant numbers and potentially compromising patient safety. As various facets of the protocol continue to be refined, the number of eligible candidates may increase and is a possibility for future study. The process of patient enrollment also highlights the selection bias that is inherent in outpatient arthroplasty, yet this is a necessary limitation. In general, younger and healthier patients with fewer comorbidities and a lower ASA score are considered acceptable candidates. An additional limitation is a lack of costing and financial analysis. While cost savings are implied due to the nature of outpatient arthroplasty and reduced length of hospital stay, and this fact has been confirmed via other studies to date, we did not set out to determine this specifically with the present study. Though this would be ideal to include in future research, costing is often difficult to achieve and, therefore, demonstrating safety of surgery performed using limited resources such as inpatient hospital infrastructure is meaningful.  While we did include a cohort group propensity matched via multiple factors, there was a significant difference in ASA score between the outpatient and inpatient groups and is likely a result of our non-randomized design. Despite the statistical significance of this, it remains unclear whether this constitutes a relevant clinical difference as the number of comorbidities was similar between groups. Finally, further limitations include a lack of randomization, and a lack of generic PROMs being utilized at all participating institutions.” |
| Interpretation | 20 | Give a cautious overall interpretation of results considering objectives, limitations, multiplicity of analyses, results from similar studies, and other relevant evidence | 11-12 | Conclusion sessoin |
| Generalisability | 21 | Discuss the generalisability (external validity) of the study results | 9 | “In our study, 61 of 68 patients (90%) were discharged safely and successfully on the same day as their surgery. This discharge rate is consistent with other studies of outpatient THA regardless of approach [10, 12, 14, 16, 18, 19, 22-29, 32].” |
| Other information | |  | | |
| Funding | 22 | Give the source of funding and the role of the funders for the present study and, if applicable, for the original study on which the present article is based | No funding |  |

*Give information separately for cases and controls in case-control studies and, if applicable, for exposed and unexposed groups in cohort and cross-sectional studies.

**Note:** An Explanation and Elaboration article discusses each checklist item and gives methodological background and published examples of transparent reporting. The STROBE checklist is best used in conjunction with this article (freely available on the Web sites of PLoS Medicine at http://www.plosmedicine.org/, Annals of Internal Medicine at http://www.annals.org/, and Epidemiology at http://www.epidem.com/). Information on the STROBE Initiative is available at www.strobe-statement.org.
